# Integrated analysis of scRNA-seq and bulk RNA-seq reveals that GPRC5A is an important prognostic gene in pancreatic cancer and is associated with B-cell Infiltration in pancreatic cancer

**DOI:** 10.3389/fonc.2024.1283164

**Published:** 2024-04-03

**Authors:** Chunlu Dong, Haidong Ma, Ningning Mi, Wenkang Fu, Jianfeng Yi, Long Gao, Haiping Wang, Yanxian Ren, Yanyan Lin, Fangfang Han, Zhou Chen, Wence Zhou

**Affiliations:** ^1^ The First School of Clinical Medicine of Lanzhou University, Lanzhou, Gansu, China; ^2^ The First Hospital of Lanzhou University, Lanzhou, Gansu, China; ^3^ Department of Surgery, The First School of Clinical Medicine of Gansu University of Chinese Medicine, Lanzhou, Gansu, China; ^4^ Lanzhou University Second Hospital, Lanzhou, Gansu, China

**Keywords:** pancreatic cancer, single-cell, immune infiltration, B cell, Gprc5a

## Abstract

**Introduction:**

Pancreatic cancer (PC) is a malignancy with poor prognosis. This investigation aimed to determine the relevant genes that affect the prognosis of PC and investigate their relationship with immune infiltration.

**Methods:**

: First, we acquired PC single-cell chip data from the GEO database to scrutinize dissimilarities in immune cell infiltration and differential genes between cancerous and adjacent tissues. Subsequently, we combined clinical data from TCGA to identify genes relevant to PC prognosis. Employing Cox and Lasso regression analyses, we constructed a multifactorial Cox prognostic model, which we subsequently confirmed. The prognostic gene expression in PC was authenticated using RT-PCR. Moreover, we employed the TIMER online database to examine the relationship between the expression of prognostic genes and T and B cell infiltration. Additionally, the expression of GPRC5A and its correlation with B cells infiltration and patient prognosis were ascertained in tissue chips using multiple immune fluorescence staining.

**Results:**

The single-cell analysis unveiled dissimilarities in B-cell infiltration between cancerous and neighboring tissues. We developed a prognostic model utilizing three genes, indicating that patients with high-risk scores experienced a more unfavorable prognosis. Immune infiltration analysis revealed a significant correlation among YWHAZ, GPRC5A, and B cell immune infiltration. In tissue samples, GPRC5A exhibited substantial overexpression and a robust association with an adverse prognosis, demonstrating a positive correlation with B cell infiltration.

**Conclusion:**

GPRC5A is an independent risk factor in PC and correlated with B cell immune infiltration in PC. These outcomes indicated that GPRC5A is a viable target for treating PC.

## Introduction

Pancreatic cancer (PC) is considered to be among the most dangerous solid tumors, ranking as the seventh most prevalent reason for cancer-related mortality globally in the year 2020. (4.7% of the total cancer deaths). Contrasted with the improvement of survival rate of other cancers, the 5-year overall survival rate for PC has barely improved (1). Up to now, only the surgery is possibly a curative treatment strategy, For patients with resectable PC, the utilization of surgery in conjunction with adjuvant systemic therapy has been shown to contribute to a longer overall survival (2). Nevertheless, owing to the lack of specific symptoms and early detection methods, only 10%-15% of patients diagnosed with PC are candidates for surgery, 80%-85% of patients have locally advanced or metastasis disease at the time of diagnosis and lose the chance for surgery and with poor 5-year survival rate fewer than 5% (3). Therefore, early detection and systemic therapy are the mainstay of improving the 5-year survival rate of individuals with PC. Currently, recommended cytotoxic therapies (such as FOLFIRINOX, gemcitabine single agents, gemcitabine + albumin-bound paclitaxel, and gemcitabine + S-1) are inefficient for advanced disease. Novel therapies emerging recently, such as target agents and immune therapies making good performance in other cancers, however, have been modestly effective in PC, largely because of an insufficient understanding of PC pathophysiology characterization and molecular mechanism.

The hallmark of PC histopathology is the complex tumor microenvironment evolving dynamically in the course of tumor progression. The tumor microenvironment contains abundant non-neoplastic components, like endothelial cells, compressed vessels, immune cells, neurons, large amounts of extracellular matrix collagen, and cancer-associated fibroblasts (CAFs) (4), Those components play a vital role in anti-tumor or tumor-promoting functions (5, 6). The patient’s prognosis and treatment effect are determined by which gain competitive advantages and crosstalk of tumor cells and compartments of tumor microenvironment. Hence, investigating the mechanism underlining the process of tumor progression is crucial for developing novel medicine. Earlier bulk RNA sequencing and transcriptomic studies divided PC into two major subtypes (‘basal-like’ and ‘classical’) based on tumor’s gene signatures. Prognosis and treatment responses differ among patients with basal-like or classical subtype, ‘classical’ subtype has increased sensitivity to mFOLFIRINOX; however, the subtype basal-like A cancers have the worst response to gemcitabine-based and mFOLFIRINOX (7–9). The identification of various subtypes according to several molecular characteristics, including pure classical, immune classical, pure basal-like, stroma activated, desmoplastic, immunological escape, immune rich, and immune exhausted, has the potential to assist with the selection of specific targeted treatments or immunotherapies since these subtypes often exhibit varying prognoses (10, 11). Thus, heterogeneity of PC makes it vital to dig for more signatures for guiding precise treatment.

Single-cell RNA sequencing (scRNA-seq) analyzing the genome information at a single-cell level provides a powerful tool for the exploration of tumor heterogeneity and identification of different subtypes. Researchers identified diverse malignant and stromal cell types in pancreases and tumor samples using scRNA-seq profiles, including two ductal subtypes that expressed different malignant genes. In addition, they uncovered the correlation between specific subsets of ductal cells and the inactivation state of tumor-infiltrating T cells. Those findings provide valuable profiles for understanding the intra-tumoral heterogeneity of PC (12). Besides the above finds, another study using sequenced 5403 cells derived from two high-grade IPMNs (HGD-IPMN), two low-grade IPMNs (LGD-IPMNs), and two patients with PC, the analysis outcomes indicate that the tumor microenvironment heterogeneity dynamically altered through the course from noninvasive dysplasia to invasive cancer, anti-tumor immune component progressively depleted while tumor-promoting immune suppressive cells infiltration increased (13). Accumulating evidence shows the advantage of scRNA-seq in exploring more subtypes or new gene signatures for precise medical treatment.

In this study, gene profile datasets were downloaded from the GEO database, and TCGA and bioinformatic analysis were used to identify the heterogeneity between PC tissue and adjacent normal pancreas and explore potentially responsible genes. First, a scRNA-seq dataset containing 16 PC tissue samples and 3 adjacent normal pancreas samples isolated from PC patients was used to find the immune heterogeneity in PC versus adjacent normal pancreas and further measure the difference level. Furthermore, we analyze the differently expressed genes (DEGs) between tumor tissue and normal pancreas at the level of single cells. Afterward, the DEGs were verified using the GEO dataset and PC data from TCGA. Univariate and LASSO Cox regression analyses were conducted to identify overall survival-related DEGs. Subsequently, a prognostic gene signature was developed using gene expression and clinical data extracted from the TCGA PAAD dataset, which was validated with an external dataset from the GEO database. Our own clinic cohort specimens were used to validate the model at the mRNA level. Finally, the online database TIMER was utilized to evaluate the association between signature genes and B-cells immune infiltration and validated by multiplex immunofluorescence staining of tissue microarray.

## Materials and methods

**Figure 1:**
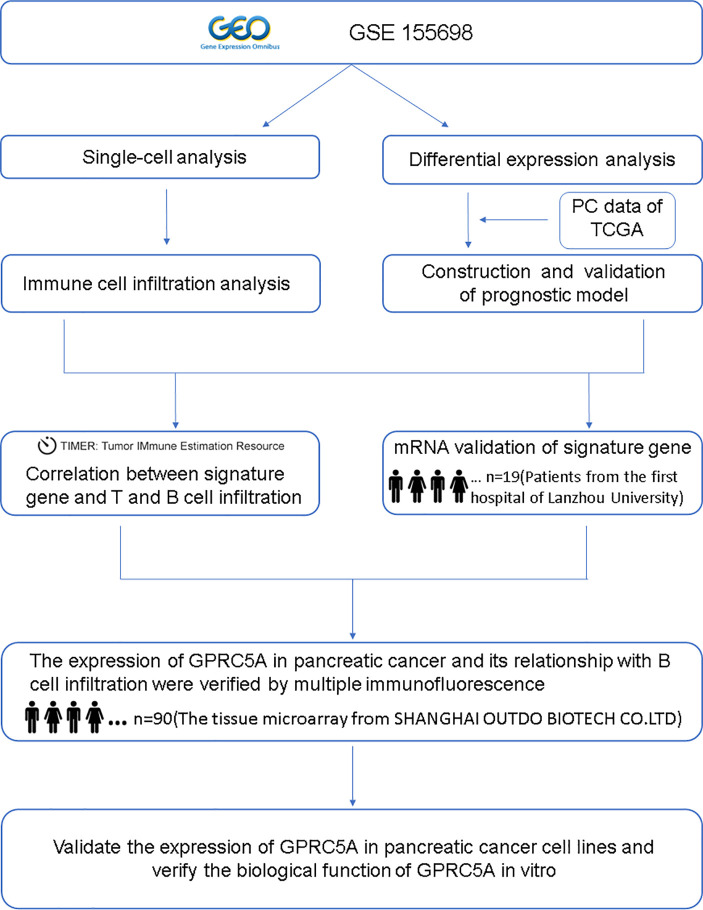
A flowchart of this study.

A flowchart of the research process is shown in **Figure 1**.

### Data acquisition

Gene Expression Omnibus (GEO) database was employed to download Single-cell transcriptome file of GSE155698 and RNA-seq files of GSE62452, GSE28735 (http://www.ncbi.nlm.nih.gov/geo/). GSE155698 consisted of 16 PC samples and 3 adjacent normal pancreas samples. The GSE62452 dataset was composed of gene expression data from 69 PC and 61 nearby healthy specimens, while GSE28735 was composed of 45 matching pairs of PC and neighboring normal tissues. PC gene expression data and clinical information were downloaded from TCGA (https://portal.gdc.cancer.gov/), and 178 PC and 4 normal healthy samples were acquired.

### Patients’ information and samples collection

19 PC tumors and paired adjacent non-tumor tissue, plus their clinic information, were collected during surgery from the first hospital of Lanzhou University, Gansu, China. Specimens were quickly placed into liquid nitrogen tanks after collection and transferred to -80°C for storage until analysis. The first Hospital of Lanzhou University’s ethical committee gives the authorization for this investigation.

### Tissue microarray information

The tissue microarray (TMA)(HPanA150Su01) was acquired from SHANGHAI OUTDO BIOTECH CO.LTD for multiplex immunofluorescence staining. The TMA contains 90 PC tissue and 60 paired adjacent non-tumor tissue from 58 male and 32 female patients. Depending on American Joint Committee on Cancer 7th cancer staging system, 37 patients are stage I, 50 patients are stage II, and 1 patient is stage IV. **Table 1** displays the fundamental data regarding the patients.

**Table 1:** Basic characteristics of 90 patients.

Variables	Total (N=90)	GRP5A<32.4 (N=35)	GRP5A≥32.4 (N=55)	P value
Age(year), mean ± SD	62 ± 11	62 ± 10	62 ± 11	0.95
Gender(male)	58 (64.44%)	27 (77.14%)	31 (56.36%)	0.045
AJCC, N(%)				0.22
Stage 1	39 (43.33%)	18 (51.43%)	21 (38.18%)	
Stage 2/3/4	51 (56.67%)	17 (48.57%)	34 (61.82%)	
T, N(%)				0.014
T1	2 (2.22%)	1 (2.86%)	1 (1.82%)	
T2	71 (78.89%)	32 (91.43%)	39 (70.91%)	
T3	17 (18.89%)	2 (5.71%)	15 (27.27%)	
N, N(%)				0.81
N0	50 (55.56%)	20 (57.14%)	30 (54.55%)	
N1	40 (44.44%)	15 (42.86%)	25 (45.45%)	
M, N(%)				1.00
M0	89 (98.89%)	35 (100%)	54 (98.18%)	
M1	1 (1.11%)	0	1 (1.82%)	
Maximum diameter of tumor (cm), mean ± SD	4.7 ± 2.0	5.2 ± 2.5	4.3 ± 1.7	0.048

### Single−cell RNA−seq analysis

The single-cell data analysis was conducted employing the “Seurat” program. We normalized and scaled the gene expression matrixes utilizing the “NormalizeData” and “ScaleData” functions. The top 2000 variable genes were selected for PCA analysis, and the 50 most significant primary components were used. Non-linear dimensional reduction was performed with the TSNE method. We further used the “Run Harmony” function to eliminate the batch effects by integrating single-cell data from numerous specimens. Cell types were annotated depending on canonical cell markers and the Human Primary Cell Atlas (HPCA) from the “SingleR” package. Differentially expressed genes (DEGs) were filtered by the principles of |log2FC|>1, p-value < 0.05. The Hallmark gene sets were obtained for Gene Set Enrichment Analysis (GSEA) from the Molecular Signatures Database.

### Development and validation of prognostic model

The TCGA PAAD dataset was used to identify overall survival-related DEGs. LASSO Cox regression is a method for variable selection and shrinkage in Cox proportional hazards model, which has been described elsewhere (14). A statistically significant outcome was seen in the univariate Cox regression analysis, as shown by a p-value of less than 0.05. LASSO Cox regression analysis with 10-fold cross-validation was employed to further reduce the number of optimal predictive DEGs. The regression coefficients of the LASSO Cox regression model were linearly combined with DEGs mRNA expression levels to construct a prognostic model for pancreatic cancer patients according to the following formula: risk score = $\sum_{i=1}^{n}\beta i\times Xi$ . Kaplan-Meier analysis, area under the curve (AUC) of the receiver operating characteristic (ROC) curve were used to evaluate the performance of the prognostic gene signature. The GSE62452 dataset was used for external validation.

### Immune cell infiltration analysis by CIBERSORT

The investigation of immune cell infiltration and the exploration of immunological microenvironment in PC were conducted employing the online tool CIBERSORT (http://CIBERSORT.stanford.edu/), depending on the standardized gene expression data from GSE62452 and GSE28735. The reference collection consisted of 22 genes associated with immune cells, known as LM22. A p-value < 0.05 in the CIBERSORT outcomes was retained. The visualization of immune cell infiltration outcomes was performed employing the “vioplot” software.

### RT-PCR

Total RNA was obtained from samples and cells according to the operation manual of TRIzol RNA extraction reagent (Invitrogen US). The concentration of RAN was measured by a Nanodrop spectrophotometer (Thermo Fisher Scientific, US), and the PrimeScript RT Master Mix (TaKaRa, JPN) was utilized for the creation of cDNA based on the protocol. RT-PCR reactions were performed according to a two-step PCR amplification procedure using a Bole PCR instrument (Bio‐Rad, US). GAPDH was selected for internal reference. The primer sequence of RT-PCR was: GAPDH, (F)5’-CCATCACCATCTTCCAGG-3’, (R)5’-ATGAGTCCTTCCACGATAC-3’, YWHAZ, (F)5’-CCTGCATGAAGTCTGTAACTGAG-3’, (R)5’-GACCTACGGGCTCCTACAACA-3’, KRT7, (F)5’-AGTATGAGGAGATGGCCAAATG-3’, (R)5’-CTGGTTCTTGATGTTGTCGATC-3’, GPRC5A, (F)5’-CTCACTCTCCCGATCCTCGT-3’, (R)5’-CAGTCCGATGATGAAGGCGAA-3’.

### Westen blotting

In the experimental procedures, cells are harvested during the logarithmic growth phase. Subsequently, total proteins were isolated from cells using the RIPA lysis buffer (CST, USA), PMSF (MCE, USA), protease inhibitors (MCE, USA). The protein concentration was determined using the bicinchoninic acid (BCA) method. Next, protein separation is achieved through electrophoresis. The isolated proteins are then transferred onto a PVDF membrane, which is blocked with 5% skimmed milk at room temperature for 1 hour. Subsequently, the membrane is incubated overnight at 4°C with primary antibodies-GPRC5A at a 1:2000 dilution and GAPDH at a 1:3000 dilution. The next day, after rewarming, the membrane undergoes incubation with secondary antibodies, specifically a goat anti-rabbit secondary antibody diluted at 1:3000, for 1 hour at room temperature. Chemiluminescence is initiated using the ECL reagent, following the provided instructions. GAPDH serves as the internal control, and ImageJ software (V1.8.0.112) is used for analyzing the relative expression of the GPRC5A protein.

### Cell culture and lentiviral transfection

Human PC cell lines (PANC-1, SW1990, BXPC-3, ASPC-1), along with the normal pancreatic cell line HPDE, were generously provided by the Key Laboratory of the Digestive System Tumors of Gansu Province (Lanzhou, China). The cells were cultured in modified Eagle’s medium (DMEM) (Gibco, USA) or RPMI-1640 (Gibco, USA) media supplemented with 10% fetal bovine serum (FBS) at 37°C in a CO_2_ incubator with 5% CO2. The human GPRC5A coding sequence and negative control (NC) sequence were synthesized and then cloned into the GV344 lentiviral vector(http://www.genechem.com.cn/service/index.php?ac=gene&at=vector_search&keyword=GV344) by GeneChem Inc. (Shanghai, China). The target sequences were as follows: sh-GPRC5A75: CCTTACAAAGACTATGAAGTA; sh-GPRC5A76: GCTTATGTTAGTCCCGAGTTT: sh-GPRC5A77: GCCCTTAATCTTGCTGTTATT; sh-NC: TTCTCCGAACGTGTCACGT. Viral transfection procedures were executed according to the provided manual, and the efficiency of transfection was evaluated using RT-PCR and Western blot analyses. Two cell lines demonstrating effective knockdown were chosen for subsequent experiments.

### CCK-8 assay

Evaluate cell proliferation using the CCK-8 method. Following a 48-hour transfection, cells are enzymatically digested, counted, and plated into 96-well plates at a density of 5,000 cells per well, with three parallel wells for each group. Subsequently, the cells are cultured for an additional 24, 48, and 72 hours. CCK-8 reagent is added to the wells at different time points as per the product instructions and incubated at 37°C for 2 hours. Absorbance at 450 nm is quantified using a Biotek microplate reader.

### Wound healing assay

Transfect PC cells (at a concentration of 2×10^5^ cells per well) into a sextuple well plate. Employ a micropipette (200 μl) to fashion a “cross” incision in each well, succeeded by lavation with phosphate buffer saline (PBS) to eliminate cellular detritus. Augment the growth medium with 10% fetal bovine serum (FBS) and capture images by inverted microscope (Olympus, Japan) at both the initiation and culmination of a 24-hour interval for subsequent quantification using ImageJ.

### Transwell assay

Appraise the invasive propensity via Transwell experimentation by adjusting the cell density of transfected PANC-1 cells to 2.5×10^5^ cells/ml post enumeration. Introduce 200 μl of cellular suspension to the superior Transwell chamber and 500 μl of medium imbued with 10% FBS to the inferior chamber. Following a 24-hour incubation period, the chambers were washed with phosphate buffered saline (PBS), fixed with 4% paraformaldehyde for 20 minutes, stained with 0.1% crystal violet for 30 minutes, subjected to three PBS rinses, air-dried, positioned under a microscope, and three random fields were selected for image capture by microscope (Olympus, Japan). Quantitative analysis was subsequently conducted using ImageJ.

### Multiplex immunofluorescence staining

According to the operation manual of Opal 7-color Guide IHC Kit (NEL801001KT, PerkinElmer), the staining steps are as follows: 1. The tissue chip was placed in a 63°C oven to dewax for 1 h, 2. then was dewaxed with xylene and gradient ethanol, 3. After antigen retrieval, a blocking buffer was added and applied for 10 min of incubation, 4. Blocking buffer was eliminated, the primary antibody was supplemented, and 1-h incubation was applied at room temperature, then the slides were washed, 5. A secondary antibody was added and incubated for 10 min at room temperature, and then the slides were washed, 6. Opal dye diluent (diluted 1:100) was added and incubated at room temperature for 10 min, and then the slides were washed, 7. After microwave treatment to remove non-covalently bound primary and secondary antibodies, the slides were washed. Steps were repeated 3 times until all labeled antibodies were applied, then a fluorescence quenching mounting medium was added to seal the slides. The Vectra Polaris, a fully computerized quantitative pathological imaging system developed by PerkinElmer, was used to scan and acquire images, and the TISSUEFAXS VIEWER software was used for image interpretation.

### Statistical analysis

Single-cell analysis, model construction, and validation were all conducted using R software (v4.2.2). Multiple immune fluorescence staining analyses and the analysis of function assay were performed using GraphPad Prism software (v8.0). Clinical data were performed with SAS Studio. All patients were classified into two groups by GRP5A expression. The cut-off value of GRP5A was determined by receiver operating characteristic (ROC) and the maximum Youden Index analyses. Group comparisons were assessed using t-tests, chi-square test or Fisher exact test, as appropriate. Spearman’s correlation coefficient was calculated to analyze the association between CD20 and GPRC5A. The Kaplan-Meier method, log-rank test, and Cox regression were used to examine the differences in survival between two groups. A p value of less than 0.05 was considered statistically significant.

## Results

### Single cell RNA−seq analysis

**Figure 2:**
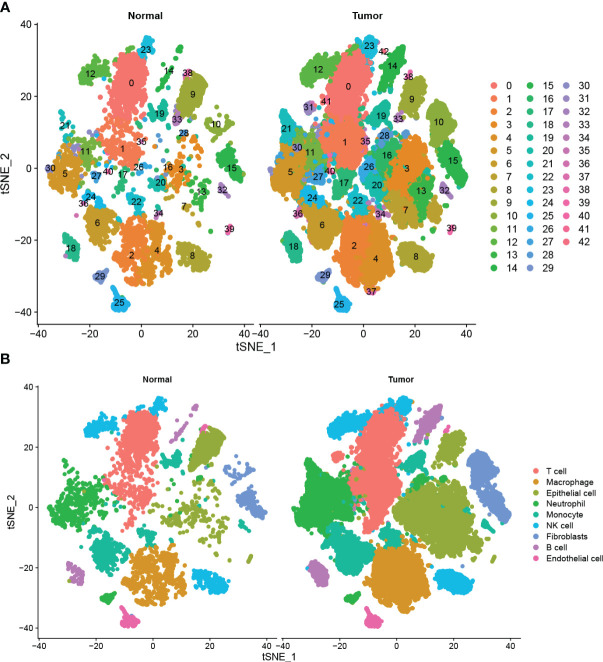
Single-cell analysis of pancreatic cancer and adjacent non-cancerous tissues **(A)** Total of 42 cell subpopulations were identified, and shown with t-SNE maps in tumor and adjacent samples, respectively. **(B)** The 42 subpopulations were re-grouped into 9 different cell types, and shown with t-SNE maps in tumor and adjacent samples.

**Figure 3:**
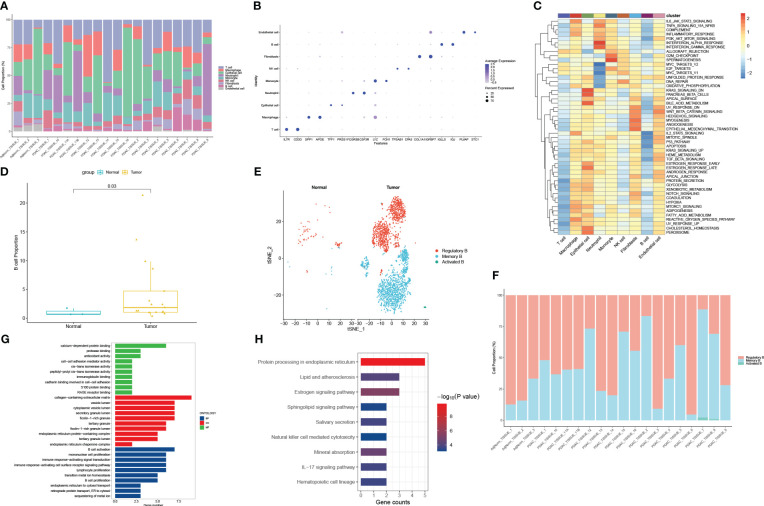
Functional and proportion of cell types analysis. **(A)** The proportion of different kinds of cells in each sample. **(B)** dot plot of the top 2 marker gene expression of subgroups. **(C)** subgroups in cancer tissue and Proportion and cell number of adjacent tissue. **(D)** B cells subtypes proportion in pancreatic cancer and normal samples. **(E)** The cellular diversity of infiltrating B cells. **(F)** The proportions of different subtypes of B cells across different samples. **(G)** Top 10 terms of GO analysis in B cell-related signature genes. **(H)** KEGG pathways analysis of B cell-related signature genes.

A total of 42 unique subpopulations were determined utilizing techniques such as data integration, scaling, and PCA dimensionality reduction (**Figure 2A**). The 42 subpopulations were categorized into nine different kinds of cells based on the immune cell biomarker gene expression (**Figure 2B**). The proportion of different kinds of cells in each sample were illustrated in (**Figure 3A**). A total of 569 cluster-specific marker genes were identified, with the expression of the top 2 genes shown in (**Figure 3B**). We presented GSEA results by heatmaps to illustrate differences in the immune microenvironment (**Figure 3C**). The further analysis revealed significant differences in B cell populations between pancreatic cancer tissues and adjacent normal pancreas (**Figure 3D**). To further dissect the cellular diversity of infiltrating B cells, we annotated B cells based on the expression of known B cell marker genes (15, 16). They included regulatory B cells, memory B cells and activated B cells (**Figure 3E**). (**Figure 3F**) shows the proportions of different subtypes of B cells across different samples. Functional analysis showed that B cell activation, B cell proliferation, and regulation of apoptotic signaling pathway was mainly enriched in B cell-related cluster (**Figures 3G, H**).

**Figure 4:**
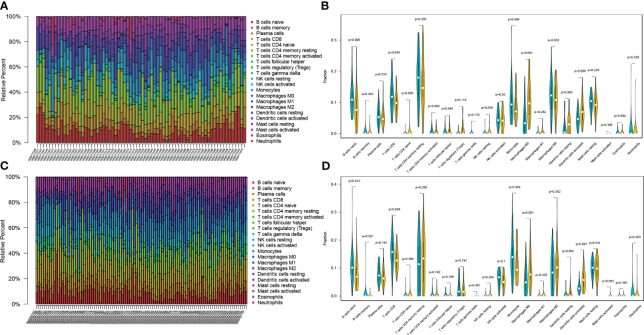
Validating the expression profile of immune cells in the GEO database **(A, B)** The expression proportion of immune cells in the GSE28735 dataset. **(C, D)** The expression proportion of immune cells in the GSE62452 dataset.

In order to identify the presence of immune cell infiltration in PC tissue and normal tissue, two additional GEO datasets were used. These datasets were submitted to the CIBERSORT algorithm, which facilitated the estimation of relative abundances of infiltrating immune cells. The findings align with those obtained through single-cell RNA sequencing analysis, as depicted in **Figure 4**. Overall, there is dysregulation of B cells in pancreatic cancer compared to normal tissues.

### Establishment and validation of the prognostic model

**Figure 5:**
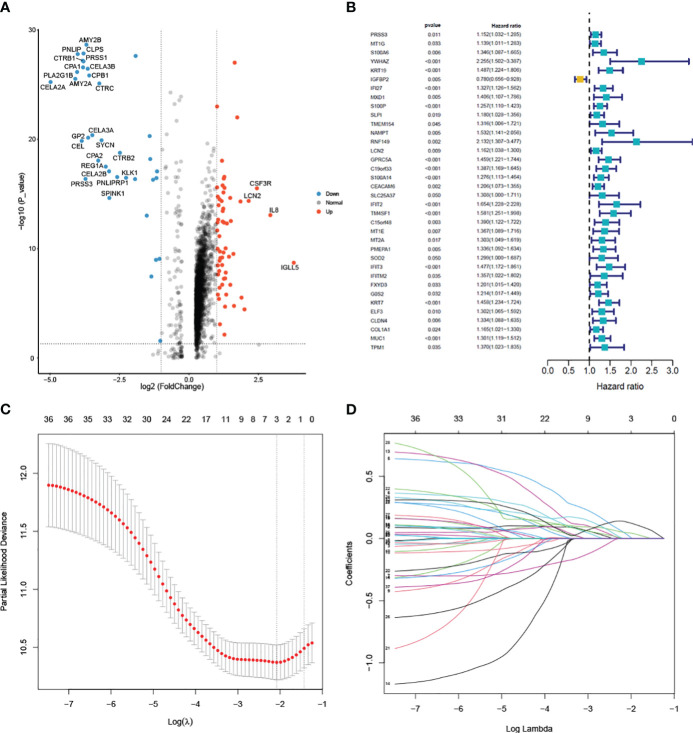
Differential analysis of single-cell data and model construction **(A)** Volcano plot of the 94 DEGs between pancreatic cancer and normal samples. **(B)** Univariate cox regression analysis of 36 prognostic genes. **(C)** Selection of the optimal parameter (lambda) in the LASSO model. **(D)** LASSO coefficient profiles of the 36 genes.

**Figure 6:**
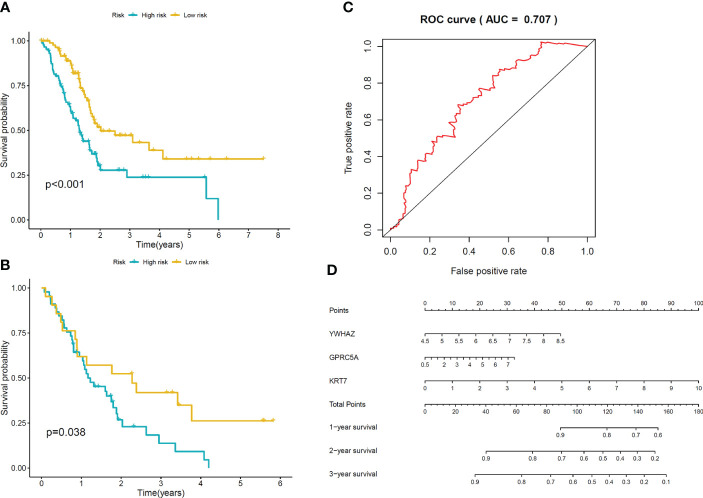
Validation of the model **(A)** Kaplan-Meier curves of OS for patients in low-risk and high-risk groups in TCGA cohort. **(B)** Kaplan-Meier curves of OS for patients in low-risk and high-risk groups in GSE62452 cohort. **(C)** The area under ROC curve was utilized to determine the predictive utility of the model. **(D)** Nomogram for predicting the overall survival of patients.

To construct the prognostic model, we identified a total of 94 Differentially Expressed Genes (DEGs) using the single-cell RNA sequencing dataset. The volcano plot illustrating these DEGs is presented in **Figure 5A**. 36 predictive genes were detected employing univariate Cox proportional hazards regression (**Figure 5B**). A prognostic signature comprising three genes, including YWHAZ, GPRC5A and KRT7 was developed by LASSO Cox analysis (**Figures 5C, D**). The model was (0.303∗ YWHAZ expression level) + (0.114∗ GPRC5A expression level) + (0.245∗ KRT7 expression level). To assess the prediction efficacy of model, we computed the AUC value of ROC. Subsequently, Kaplan-Meier survival analysis was performed. The AUC value of model for overall survival (OS) was 0.707 (**Figure 6C**). The individuals in the high group had significantly worse survival outcomes contrasted to those in the low group (**Figure 6A**). We also performed a nomogram (**Figure 6D**). To validate the novel model capability, the formula defined above was conducted to examine the GSE62452 dataset. Kaplan-Meier survival analysis outcomes were consistent with the TCGA analysis (**Figure 6B**). These findings suggest that the prognostic model constructed using single-cell RNA sequencing data demonstrates favorable predictive value.

### Validation of signature genes at mRNA level in our cohort

**Figure 7:**
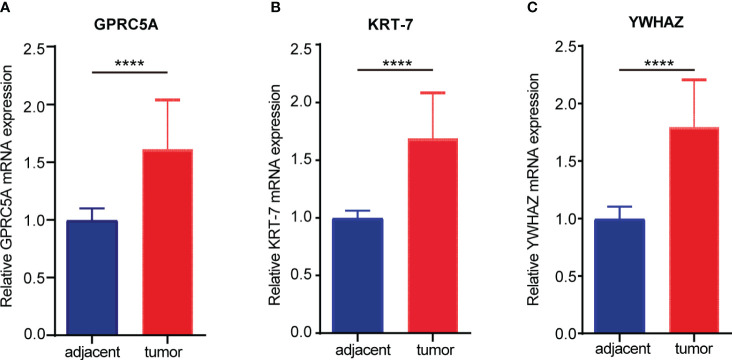
The mRNA expression of signature genes in the cohort from the first hospital of Lanzhou University. **(A)** The expression of GPRC5A in pancreatic cancer and adjacent tissues. **(B)** The expression of KRT7 in pancreatic cancer and adjacent tissues. **(C)** The expression of YWHAZ in pancreatic cancer and adjacent tissues. (****P < 0.0001).

To validate the mRNA expression of signature genes, RT-PCR was performed in 19 paired PC and adjacent normal pancreas samples; the result demonstrated that three signature genes are significantly higher expressed in PC tissue than in adjacent normal pancreas samples (p< 0.0001, **Figure 7**).

### Relationship between signature genes and immune cell infiltration in PC

**Figure 8:**
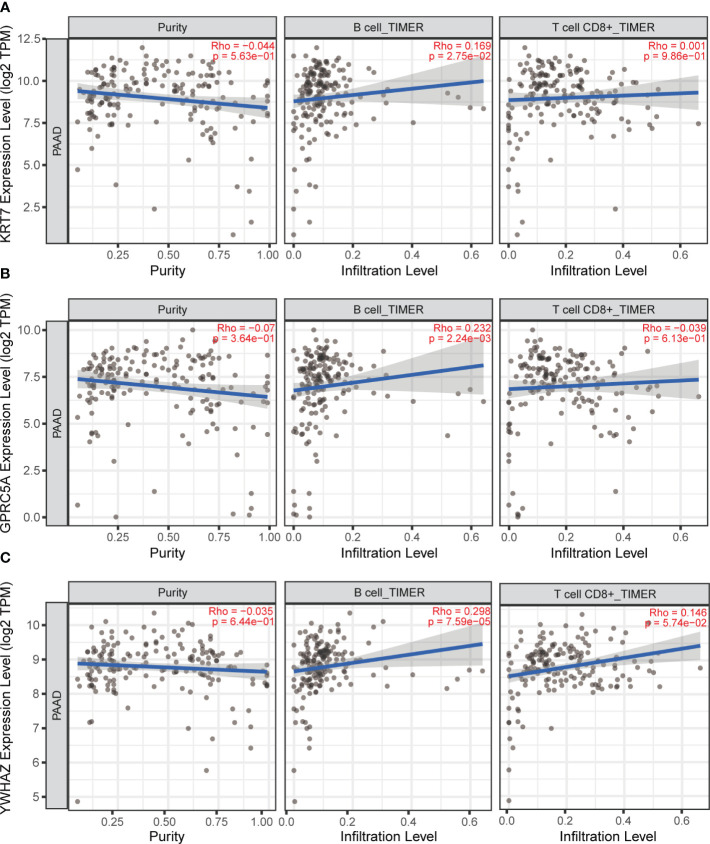
TIMER online database was used to analyze the correlation between KRT7, YWHAZ and GPRC5A gene expression and T and B cell infiltration in pancreatic cancer. **(A)** The correlation between KRT7 and T-cell, B-cell. **(B)** The correlation between GPRC5A and T-cell, B-cell. **(C)** The correlation between YWHAZ and T-cell, B-cell.

Immune cell infiltration is a pivotal component of the tumor microenvironment, significantly influencing tumor initiation, development, metastasis, and the effectiveness of immune-targeted therapies. In this investigation, we aimed to explore the relationship between three model genes in PC and the infiltration of major immune cells. For this purpose, we employed the TIMER online database to study the expression levels of KRT7, YWHAZ, and GPRC5A genes and their correlation with T cells and B cells in PC. Our comprehensive correlation analysis reveals a positive connection between the KRT7, YWHAZ, and GPRC5A gene expression and the infiltration of B cells in PC (**Figure 8**). Interestingly, our results reveal that, in contrast to KRT7, the expression of YWHAZ and GPRC5A genes demonstrates a relatively stronger correlation with B cell infiltration in pancreatic cancer.

### The protein expression of GPRC5A in PC and its relationship with prognosis and B-cell infiltration

**Figure 9:**
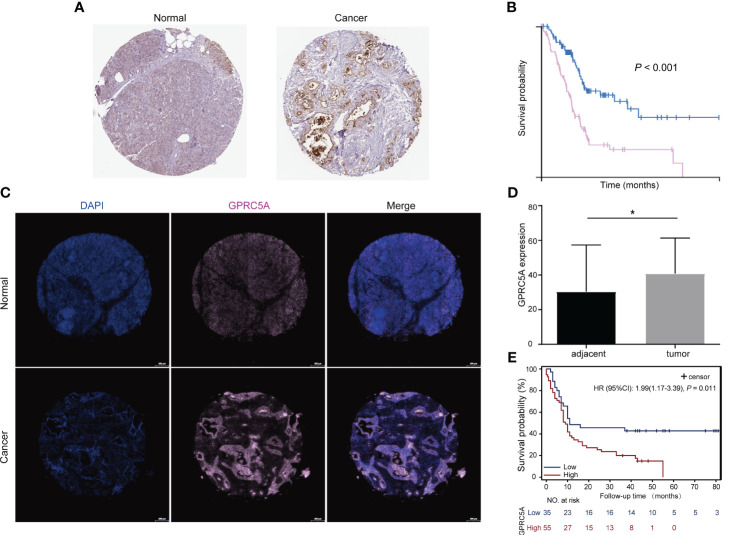
Protein expression of GPRC5A in pancreatic cancer and adjacent tissues and its relationship with patient prognosis **(A)** GPRC5A expression stained in pancreatic cancer and adjacent tissues, data from The Human Protein Atlas (HPA) database. **(B)** IHC showed that patients with high GPRC5A expression had a shorter survival time, data from HPA database. **(C, D)** Multiplex immunofluorescence staining showed that GPRC5A was highly expressed in pancreatic cancer tissues in TMA. **(E)** Patients with high GPRC5A expression had a shorter survival time in TMA cohort. "*" means p<0.05.

**Figure 10:**
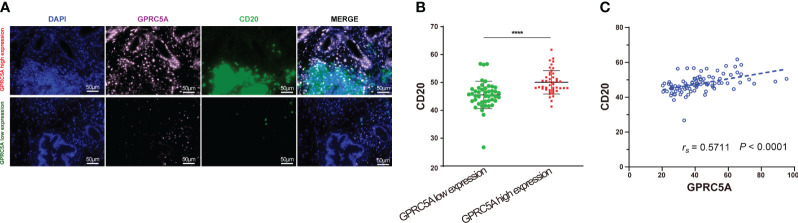
Multiplex immunofluorescence staining of GPRC5A and CD20 in TMA **(A, B)** Multiplex immunofluorescence staining showed patients with high GPRC5A fluorescence values had high CD20 fluorescence values. **(B)** Correlation between GPRC5A and CD20. "****" means p<0.0001.

The G-protein-coupled receptor family (GPCRs) constitutes the largest membrane receptor superfamily in humans, participating in a wide range of physiological and pathological processes while also possessing accessible drug-binding sites on the cell surface, rendering them attractive potential drug targets. Notably, GPRC5A has been identified as a significant prognostic gene in various PC prognostic models (17, 18). To delve deeper into the protein expression level of GPRC5A, we conducted a thorough search in the HPA database, revealing that, contrasted to healthy pancreatic tissues, GPRC5A was remarkably overexpressed in PC tissues (**Figure 9A**). Moreover, survival analysis demonstrated that patients with elevated GPRC5A expression exhibited longer survival times (**Figure 9B**). To validate our findings, we performed multiple immune fluorescence staining on tissue microarrays generated from 90 PC patient samples. These experiments further substantiated the heightened expression of GPRC5A in PC tissues (**Figures 9C, D**), with its expression level showing an association with patient survival (**Figure 9E**). Univariate and multivariable COX regression analyses (**Table 2**) independently identified GPRC5A as a significant risk factor for PC prognosis. Additionally, our research reveals a strong correlation between high GPRC5A expression in pancreatic cancer tissues and elevated levels of CD20^+^ B cell infiltration (r_s_ = 0.5711, p < 0.0001, **Figure 10**).

**Table 2:** Univariable and multivariable Cox regression analysis of GPRC5A expression and other covariates on survival of PC patients.

Characteristics	Univariate analysis		Multivariate analysis	
	HR (95%CI)	P	HR (95%CI)	P
Age(year)	1.02 (0.99-1.04)	0.15		
Gender				
female	1.00			
male	1.16 (0.70-1.93)	0.56		
AJCC, N(%)				
Stage 1	1.00		1.00	
Stage 2/3/4	1.82 (1.10-3.00)	0.02	1.66 (1.00-2.76)	0.049
Maximum diameter of tumor (cm)	0.99 (0.87-1.13)	0.91		
GRP5A				
low	1.00		1.00	
high	1.99 (1.17-3.39)	0.01	1.84 (1.07-3.15)	0.027

### The impact of GPRC5A on the proliferation, migration, and invasion of PC cells

**Figure 11:**
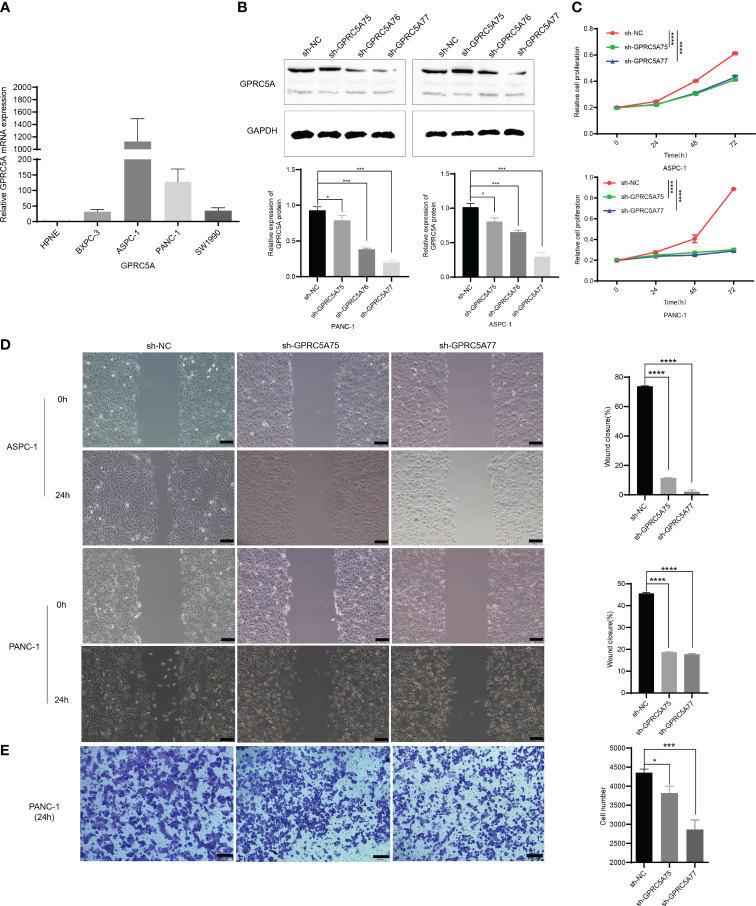
GPRC5A promotes PC cells proliferation, migration, and invasion **(A)** The mRNA expression of GPRC5A in PC cells. **(B)** The validation of knockdown efficiency through Western blot analysis. **(C)** The CCK-8 experiment confirming the influence of GPRC5A on the proliferation of PC cells. **(D)** The results of the scratch assay. **(E)** The Transwell invasion assay results for the PANC-1 cell line. (*P < 0.05, **P < 0.01, ***P < 0.001, ****P < 0.0001).

To ascertain the expression of GPRC5A in PC cells, we conducted an analysis of its mRNA expression in both normal pancreatic cells (HPDE) and PC cells (PANC-1, SW1990, BXPC-3, ASPC-1). The findings indicated elevated GPRC5A expression in PC cell lines (**Figure 11A**). Subsequently, two PC cell lines (PANC-1, ASPC-1) exhibiting high GPRC5A expression were chosen for further investigation based on the mRNA results. After lentiviral transfection, the knockdown efficiency was assessed through RT-PCR and Western blot analyses, the results demonstrated a significant decrease in both mRNA (P < 0.05, **Supplementary Figure S3**) and protein expression (P < 0.05, **Figure 11B**) in the sh-GPRC5A group compared to the sh-NC group.

CCK-8 assays were employed to evaluate cell proliferation, indicating a significant inhibition in PANC-1 and ASPC-1 cells in the sh-GPRC5A group compared to the sh-NC group (p < 0.0001, **Figure 11C**). Wound healing assays were conducted to investigate the impact of GPRC5A on migration ability in PC cells, revealing that GPRC5A knockdown led to reduced wound closure compared with the sh-NC group (p < 0.0001, **Figure 11D**). Transwell experiments were performed to assess invasive capabilities, illustrating a significant inhibition in PANC-1 cells with GPRC5A knockdown (P < 0.05, **Figure 11E**). Taken together, these outcomes collectively suggest that GPRC5A knockdown suppresses both the proliferation, migration, and invasion of PC cells.

## Discussion

PC is a fatal malignancy disease with a poor overall 5-year survival rate of 12% (19). Since most patients are unsuitable for surgery, medical treatment has become an important approach to improve the prognosis of PC. Over the past few decades, chemotherapy, targeted therapy, and immunotherapy have shown great potential in cancer treatment. However, due to the heterogeneity and complex tumor microenvironment of PC, the effectiveness of these therapies is limited in PC. Recently, research based on gene expression profiling can identify subtypes with unique biological and clinical characteristics, allowing for tailored treatment strategies for different tumor subtypes. Therefore, the search for new biomarkers is of great significance for optimizing clinical decision-making.

ScRNA-seq analysis provides a tool for precisely exploring potential target genes for drug development (20). In this study, we combined scRNA-seq data with TCGA data to construct a new prognostic model containing three genes (KRT7, YWHAZ, and GPRC5A). External validation demonstrated the stable predictive capability of this model. In our patient cohort, KRT7, YWHAZ, and GPRC5A were significantly upregulated in PC tissues, further validating the reliability of model. Additionally, studies have reported that inhibiting KRT7 transcription leads to the inactivation of PI3K/AKT signaling mechanism, thereby delaying the progression of PC (21). Increased expression of 14-3-3ζ protein encoded by the YWHAZ gene inhibits the degradation of β-catenin, leading to upregulation of β-catenin-dependent gene expression, thus promoting glycolysis and enhancing PC cell growth and invasion capabilities (22). GPRC5A is a potential oncogene in adenocarcinoma cells of the pancreatic duct, and knocking out GPRC5A can reduce the proliferation and migration ability of PC cell lines, as well as inhibit their chemoresistance to gemcitabine, oxaliplatin, and fluorouracil (23). Remarkably, among 675 human cancer cell lines, the mRNA expression of GPRC5A in PC cell lines exhibited the highest level. In a study by Xiao et al., the analysis of DNA methylation-driven genes (MDGs) in PC revealed that elevated GPRC5A expression is associated with a poor prognosis and negatively correlates with patients’ Disease-Free Survival (DFS). Similarly, Wei et al. conducted an analysis of cDNA expression profiles from 98 PC patients and 71 normal tissues sourced from 5 different GEO datasets, revealing a significant overexpression of GPRC5A in PC tissues. Furthermore, patients exhibiting high GPRC5A expression levels demonstrated inferior overall survival and relapse-free survival rates. Collectively, these findings suggest a pivotal role for GPRC5A in PC (17, 18, 24). Hence, the current investigation focused on GPRC5A’s role in PC.

GPRC5A is a G-protein-coupled receptor (GPCR) family member and is abnormally expressed in various tumors. Multiple studies have indicated that GPRC5A serves as an important tumor suppressor gene in lung cancer. Loss of GPRC5A leads to the occurrence of lung cancer. Further research has revealed that its loss causes dysregulation of MDM2 expression through the EGFR signaling pathway, contributing to lung cancer development (25). In colorectal cancer, GPRC5A promotes tumor formation by regulating NF-κB-mediated Vanin-1 expression and oxidative stress (26). GPRC5A was found to be downregulated in triple-negative breast cancer (TNBC), and its overexpression induced cell apoptosis by regulating the PI3K/Akt signaling pathway, suggesting that GPRC5A serves as a protective factor against TNBC progression by enhancing apoptosis (27).. In PC, our multiple immunofluorescence experiments have confirmed a direct correlation between GPRC5A and the OS of individuals with PC. GPRC5A is an independent risk factor influencing the survival of PC, according to univariate and multivariate COX regression analyses. Moreover, *in vitro* experiments also confirmed that GPRC5A expression promotes proliferation, migration and invasion of pancreatic cancer cells. Therefore, subjects with PC and high GPRC5A expression exhibit shorter survival and poorer prognosis, making it a reliable indicator for prognostic assessment in PC patients.

The immune infiltration in PC has a pivotal role in tumor progress and significantly impacts clinical outcomes for cancer patients. Among immune cells, T cells and B cells emerge as key players, each assuming distinct and critical roles. T cells’ function in anti-tumor immunity is well established. However, mounting evidence suggests that tumor-infiltrating B cells exert vital synergistic effects in inhibition of tumor growth, they facilitate antigen presentation, effectively triggering T cell production and promoting the generation of anti-tumor antibodies, thus fostering anti-tumor responses (28, 29). Additionally, B cells contribute to tumor development and hinder T cell-mediated anti-tumor immune responses by selectively activating macrophages. Remarkably, the depletion of B cells in tumor-bearing mice led to reduced PanIN progression and decreased invasive cancer incidence (30). Certain subsets of B cells secrete growth factors and immunosuppressive factors that favor tumor cell growth, allowing tumor cells to evade immune surveillance and drive tumor progression (31). Furthermore, the growth of PC tumors is contingent upon the intricate interplay between B cells and tumor-associated macrophages expressing FcRγ. This dynamic interaction leads to the polarization of macrophages into a TH2-type phenotype, facilitated by the activation of Bruton’s tyrosine kinase (BTK) in a manner dependent on phosphatidylinositide 3-kinase (PI3K)γ (32). Studies indicate that B cell receptor activation regulates B cell-derived IL-35 expression through downstream protein kinase D2 (PDK2), suppressing the immune capacity of anti-tumor T cells and fostering PC development. Inhibition of B cell-derived IL-35 production enhances PC sensitivity to PD-1 immune checkpoint inhibitors, offering potential therapeutic implications (33, 34).We conducted bioinformatics analysis, and the results revealed that B-cell infiltration in PC tissues was higher than that in adjacent normal pancreas.

The interplay between cancer and immune cells determines the magnitude of immune reaction and the tumor cells’ outcome. For example, during tumor progression, tumor cells continuously uptake nutrients to facilitate rapid proliferation, and this metabolic shift may alter the survival and function of immune cells, ultimately leading to immune escape and tumor progression. A study involving co-cultures of tumor cells with T lymphocytes demonstrated that with increasing tumor cell density, the production of IFN-γ by CD4+ T cells significantly decreased, while the expression level of PD-1 in CD4+ T cells elevated with the growing number of tumor cells in the co-culture system (35). It is well known that the PD-1/PD-L1 signaling pathway is a crucial mechanism for tumor immune escape. Recent research indicates that PC cells secrete IL-1α to stimulate tumor-promoting fibroblast infiltration through paracrine signaling (36, 37). IL-1β is extensively expressed in cell lines and surgical specimens of PC (38), and it is related to poor prognosis in individuals with PC (39, 40). Further studies using the KC-IL1β mouse model, which overexpresses IL-1β, revealed that IL-1β induces the expansion of immunosuppressive B cells by upregulating CXCL13 to promote PC development (41). Other research also suggests that IL-1β secreted by pancreatic tumors induces the infiltration of various immunosuppressive cells (42). However, the relationship between GPRC5A and B cell immune infiltration in PC has not been studied yet. Our research shows a high expression of B cells in PC tissues, and GPRC5A is positively correlated with B cell immune infiltration in PC tissues. We speculate that GPRC5A may promote the infiltration of B cells in PC tissues.

In this study, we employed scRNA-seq and bulk RNA-seq to screen and establish a prognostic model centered on three genes, which demonstrated commendable predictive ability. Moreover, we detected the signature gene expression levels in clinical samples and examined their association with T and B cells. Nevertheless, it is essential to expand the sample size in future experiments to thoroughly ascertain the model’s effectiveness, thus furnishing valuable references for clinical treatment decisions. Additionally, while this investigation successfully uncovered the correlation between GPRC5A and B cells in tissue samples, a more comprehensive exploration of the underlying mechanisms is needed. To address this, we plan to undertake *in vivo* and *in vitro* experiments, delving deeper into the interaction between GPRC5A and B cells within the context of PC. This approach will elucidate how GPRC5A influences the immune microenvironment of PC, thereby shedding light on its specific mechanisms.

## Data availability statement

The original contributions presented in the study are included in the article/**Supplementary Material**. Further inquiries can be directed to the corresponding author.

## Ethics statement

The studies involving humans were approved by the ethics committee of the First Hospital of Lanzhou University; the ethics committee, Outdo Biotech (Shanghai, China). The studies were conducted in accordance with the local legislation and institutional requirements. The participants provided their written informed consent to participate in this study.

## Author contributions

CD: Data curation, Writing – original draft, Writing – review & editing. HM: Data curation, Writing – review & editing. NM: Data curation, Formal analysis, Methodology, Visualization, Writing – review & editing. WF: Data curation, Investigation, Writing – review & editing. JY: Visualization, Writing – review & editing. LG: Visualization, Writing – review & editing. HW: Data curation, Methodology, Visualization, Writing – review & editing. YR: Writing – review & editing. YL: Visualization, Writing – review & editing. FH: Validation, Writing – review & editing. ZC: Writing – review & editing. WZ: Conceptualization, Project administration, Writing – review & editing.
