# Supplementary figures and images for: Integrated analysis of scRNA-seq and bulk RNA-seq reveals that GPRC5A is an important prognostic gene in pancreatic cancer and is associated with B-cell Infiltration in pancreatic cancer
Source: Front Oncol. 2024 Apr 3;14:1283164. doi: 10.3389/fonc.2024.1283164 (PMC11021786; doi:10.3389/fonc.2024.1283164)

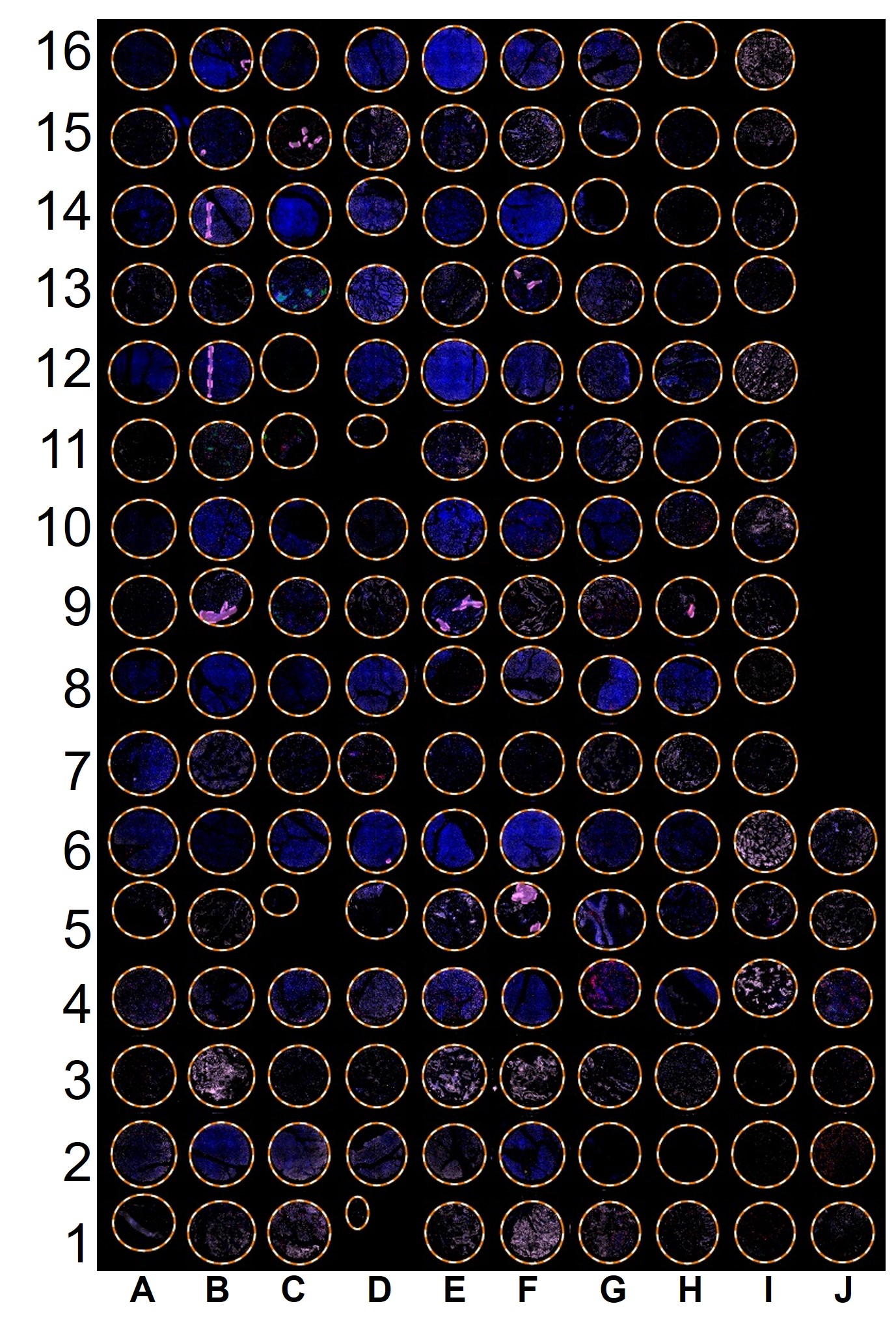

Supplement: Supplementary file 1 [file Image_1.jpeg]

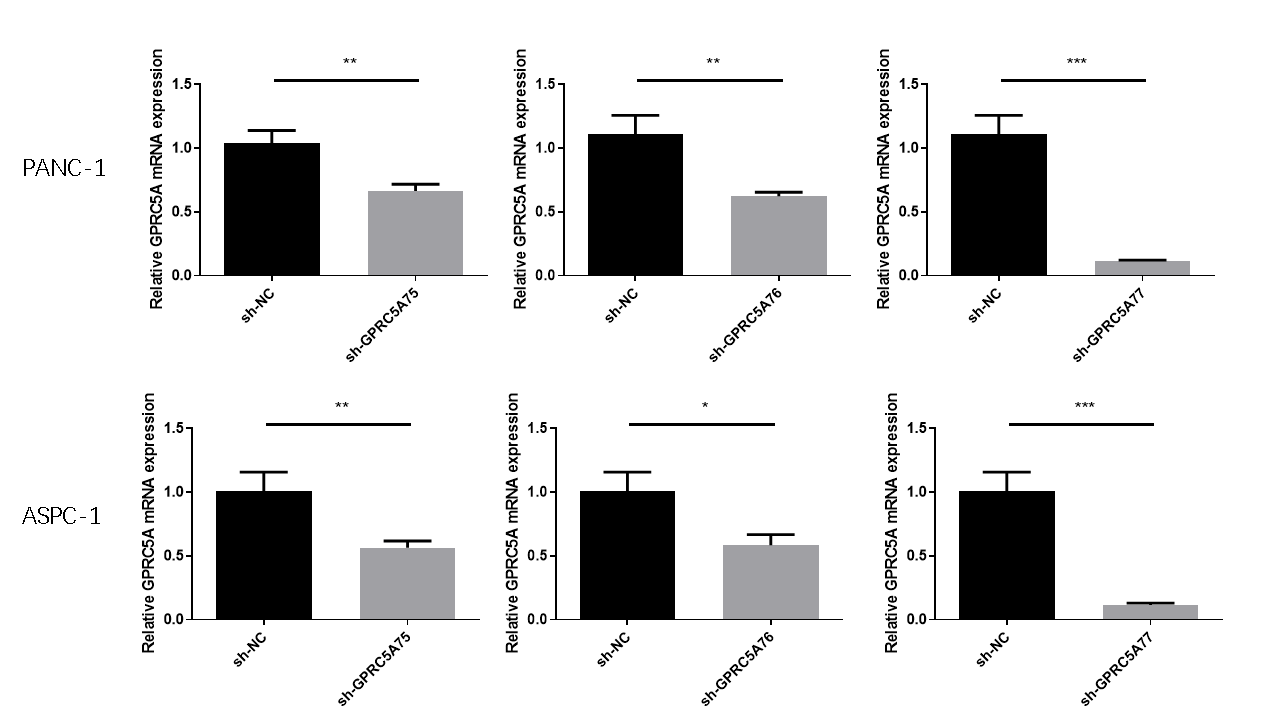

Supplement: Supplementary file 2 [file Image_2.tif]
